# Environmental Particulate Matter Induces Murine Intestinal Inflammatory Responses and Alters the Gut Microbiome

**DOI:** 10.1371/journal.pone.0062220

**Published:** 2013-04-24

**Authors:** Lisa Kish, Naomi Hotte, Gilaad G. Kaplan, Renaud Vincent, Robert Tso, Michael Gänzle, Kevin P. Rioux, Aducio Thiesen, Herman W. Barkema, Eytan Wine, Karen L. Madsen

**Affiliations:** 1 Department of Medicine, University of Alberta, Edmonton, Alberta, Canada; 2 Department of Medicine, University of Calgary, Calgary, Alberta, Canada; 3 Environmental Health Directorate, Health Canada, Ottawa, Ontario, Canada; 4 Department of Agricultural, Food, and Nutritional Science, University of Alberta, Edmonton, Alberta, Canada; 5 Department of Lab Medicine and Pathology, University of Alberta, Edmonton, Canada; 6 Department of Production Animal Health, University of Calgary, Calgary, Alberta, Canada; 7 Department of Pediatrics, University of Alberta, Edmonton, Alberta, Canada; Charité-University Medicine Berlin, Germany

## Abstract

**Background:**

Particulate matter (PM) is a key pollutant in ambient air that has been associated with negative health conditions in urban environments. The aim of this study was to examine the effects of orally administered PM on the gut microbiome and immune function under normal and inflammatory conditions.

**Methods:**

Wild-type 129/SvEv mice were gavaged with Ottawa urban PM_10_ (EHC-93) for 7–14 days and mucosal gene expression analyzed using Ingenuity Pathways software. Intestinal permeability was measured by lactulose/mannitol excretion in urine. At sacrifice, segments of small and large intestine were cultured and cytokine secretion measured. Splenocytes were isolated and incubated with PM_10_ for measurement of proliferation. Long-term effects of exposure (35 days) on intestinal cytokine expression were measured in wild-type and IL-10 deficient (IL-10^−/−^) mice. Microbial composition of stool samples was assessed using terminal restriction fragment length polymorphism. Short chain fatty acids were measured in caecum.

**Results:**

Short-term treatment of wild-type mice with PM_10_ altered immune gene expression, enhanced pro-inflammatory cytokine secretion in the small intestine, increased gut permeability, and induced hyporesponsiveness in splenocytes. Long-term treatment of wild-type and IL-10^−/−^ mice increased pro-inflammatory cytokine expression in the colon and altered short chain fatty acid concentrations and microbial composition. IL-10^−/−^ mice had increased disease as evidenced by enhanced histological damage.

**Conclusions:**

Ingestion of airborne particulate matter alters the gut microbiome and induces acute and chronic inflammatory responses in the intestine.

## Introduction

Particulate matter (PM) is a key pollutant in ambient air that has been associated with negative health conditions in urban environments [1], [2]. PM is made up of both coarse (PM_10_: Diameter <10 µm) and fine particles (PM_2.5_: Diameter <2 µm) and arises from vehicle exhaust, industrial emissions, road dust, and windblown soil [3], [4]. PM contains a complex mixture of metals, ions, and polycyclic aromatic hydrocarbons (PAH), as well as numerous biological components, including lipopolysaccharide [1], [3], [5], [6]. Epidemiological studies have shown a strong association between PM exposure and adverse health effects, including stroke, myocardial infarction, arrhythmia, cardiac arrest, venous thrombosis, and lung cancer [2], [3], [4], [7], [8], [9]. All-cause mortality has been shown to increase by 0.5% for each 10 µg/m^3^ rise of PM_10_ [10]. Emerging evidence suggests PM exposure can also have adverse consequences on the gastrointestinal tract [11], with associations shown between air pollution exposure and an increased risk of appendicitis [12], gastroenteritis [13], Crohn’s Disease (CD) in younger individuals [14], hospitalizations in patients with inflammatory bowel disease (IBD) [15], and colon and liver cancer [16].

Research on airborne pollutants has for the most part focused on the respiratory effects after inhalation as this is considered the primary route of PM exposure; however the gut is also exposed to high concentrations as the particles are removed from the lungs by mucocilliary transport and cleared via the gastrointestinal tract [17]. Intestinal exposure also occurs through the ingestion of PM contaminated water and foods [18], [19]. Recent studies have linked changes in the gut microbiome with human disease but effects of PM on the gut microbiome are largely unknown. In addition, studies have shown that PM can increase epithelial permeability through the induction of oxidative stress [20]. Increases in gut permeability have been associated with numerous diseases, including inflammatory bowel disease, diabetes, and celiac disease [21]. The interleukin-10 knockout (IL-10^−/−^) mouse is a commonly used model of spontaneous, microbial-induced colonic inflammation similar to IBD [22]. This mouse strain remains healthy when kept sterile, but develops severe colitis in the presence of a normal colonic microbiota [23]. Increases in small intestinal permeability are associated with development of colonic disease in this model and further, preventing the increase in small intestinal permeability attenuates disease [24], [25], [26]. Thus, we hypothesized that exposure of IL-10^−/−^ mice to PM would cause an increase in gut permeability, alter the gut microbiome, and exacerbate disease.

## Materials and Methods

### Particulate Matter (PM_10_)

Ambient air particulate matter (PM_10_: EHC-93) was obtained from the videlon bag filters of the single pass air-purification system from the Environment Health Center in Ottawa, Ontario, Canada. Characteristics of the PM_10_ have been previously described [3] and are summarized in Table S1.

### Animal Model

The protocol for use of mice was approved by the Health Science Animal Care and Use Committee at the University of Alberta. In order to maintain a constant environment and to reduce stress-induced effects due to shipping, wild-type (WT) and IL-10^−/−^mice on a 129 Sv/Ev background were maintained in colonies at the University of Alberta. Mice were kept in standard housing conditions of 23°C, 45% humidity and had a 12 hour light/dark cycle. In that changes in habitat can significantly alter gut microbiota [27], individual mice were maintained in conventional housing for 2 weeks prior to entering into the study. In addition, in order to try to mitigate family and cage effects, pups from the same litter were split into different treatment groups and housed with 2 animals per cage with cage-mates from different litters.

### Animal Studies – Short-term Treatment

Fasted female WT mice (6–8 wks of age) were gavaged daily in mid-afternoon with vehicle or PM_10_ (EHC-93: 18 µg/g/day) for 7 or 14 days. PM was suspended in water and shaken before each gavage to ensure even disbursement. This concentration represents a high dose that could occur during periods of extremely high levels of air pollution [28].

#### Mucosal cytokine secretion

At sacrifice, segments of the small and large intestine were cultured in RPMI 1640 (10% heat inactivated fetal bovine serum, streptomycin (1 g/ml), L-glutamine (2 mM), 2 mercapto-ethanol (50 uM), and non-essential amino acids (10 mM) (Invitrogen) for 8 hr at 35°C. Cytokine levels in the supernatant were measured using a MesoScale Discovery Kit as per manufacturer’s instructions. IL-17 was measured by ELISA (R&D systems).

#### Gene expression and pathway analysis

Mice were sacrificed at baseline, and after 7 and 14 days of treatment with PM_10_. Segments of small intestine and colon were removed and snap-frozen at −80C. RNA isolation, cDNA synthesis and relative gene expression analysis was completed as previously described [29]. Briefly, total RNA was isolated using a TRIzol extraction procedure and further purified using RNeasy columns with DNAse treatment (Qiagen, USA) followed by cDNA synthesis (Applied Biosystems). For the gene expression studies, there were a total of 4 mice per group for a biological replicate of 4. Each of the PCR reactions was completed in triplicate, for a technical replicate of 3. Relative gene expression was used to calculate the fold change expression between PM_10_ treated mice with vehicle treated. Real-time PCR based reactions were measured using 96- plex mouse immune Taqman Low Density Arrays (TLDA) (Applied Biosystems) and fold differences calculated using DataAssist software (Applied Biosystems). A geometric mean of *gusb, gapdh* and *18s* CT values was used to normalize all RT-PCR samples. These endogenous controls were chosen as they showed the greatest stability across all samples. The normalization method was calculated using a standard technique in Data Assist software v3.01 [30]. Ingenuity Pathway Analysis (IPA) software (http://www.ingenuity.com) was used to identify specific gene network interactions.

#### *In vivo* permeability

Mice in the short-term study receiving daily gavage of PM_10_ were assessed for small intestinal permeability at baseline, 7 and 14 days. Mice were gavaged with 0.2 ml of a sugar probe (6 g/ml lactulose and 4 g/ml mannitol) and housed individually in metabolic cages for 22 hrs [26]. Urine was collected in containers containing 100 µL of 10% thymol (1 g/10 mL isopropanol) and 100 µL of paraffin oil. To reduce effects of stress at being placed in the cases on gut permeability measurments, mice were placed in the metabolic cages 3 times prior to the baseline measurements. Lactulose/mannitol concentrations were quantified by ion exchange high-performance liquid chromatography (HPLC) as previously described [31]. Briefly, cellobiose was added as an internal standard, and the urine was filtered through a 0.4 µm filter and diluted as necessary. Samples were deionized and then injected on a Dionex MA-1 ion exchange column (Dionex, Sunnyvale, California, USA). Sugars were eluted with NaOH at a flow rate of 0.4 ml/min. Peaks were detected using pulsed amperometric detection on a Dionex HPLC and quantified as peak areas. Final data were reported as a ratio of fractional excretions (lactulose/mannitol). Fractional excretion is defined as the fraction of the gavaged dose recovered in the urine sample.

#### Cell isolation and proliferation

Spleens were removed, homogenized, then passed through a 70 µm nylon strainer (BD Bioscience) and centrifuged at 200 g for 5 min. Cell pellets were suspended in PBS and red blood cells lysed by adding 9 parts water followed by the addition of one part 10X PBS. Splenocytes were centrifuged at 200 g for 5 min, then suspended in Imag™ buffer (PBS+BSA +0.09% Sodium Azide). Cells were counted with a Beckman Coulter counter, diluted in RPMI media, and plated at a concentration of 1×10^5^ cells/well. Isolated splenocytes were incubated with RPMI 1640 media ± PM_10_ (0.5 mg/ml). αCD3 was used as a positive control. After 48 hrs, cell cultures were centrifuged at 1000 rpm, suspended in media containing ^3^H thymidine and incubated for 24 hr. Cells were harvested using an Inotech cell harvester and read on WallacMicrobeta Tilux Scintillation counter. Results are displayed as a ratio to αCD3 to normalize the data.

### Animal Studies – Chronic Treatment

Six week old female WT and IL-10^−/−^ mice were fed mouse chow ± PM_10_ (0.09 gm/kg) for 35 days. The standard mouse chow was recreated from powdered Lab Diet 5001 (LabDiet) plus or minus the addition of 0.09 g/kg of PM_10_. PM_10_ was added to the powder and allowed to disperse by mixing with a kitchen mixer for 10 min. Water was then added to enable reformation and the pellets were dehydrated at room temperature overnight. Preliminary experiments were carried out to determine the amount of chow consumed/day and if PM_10_ in the chow altered food consumption. Results showed that the amount of chow consumed on a daily basis by a single mouse averaged ∼3 grams, and the addition of PM_10_ did not alter the amount of food consumed (data not shown); thus mice in the study consumed ∼270 µg/day, which would be ∼10–13 µg/g/day per mouse. In IL-10^−/−^ mice in our animal colony, colitis begins to develop between 8–12 weeks of age [32]. In these studies, treatment was begun prior to the onset of inflammation to determine if PM_10_ would alter the normal development of colitis. At sacrifice, the intestine was removed and sections prepared for histology or homogenized for cytokine expression. Stool samples were analyzed for microbial composition. For histology, intestinal sections were fixed in 10% phosphate-buffered formalin, paraffin-embedded, then sectioned and stained with haematoxylin and eosin for microscopic examination. Slides were reviewed in a blinded fashion by a pathologist (AT) and assigned a histologic score [32].

#### Short chain fatty acid analysis

Short chain fatty acid concentrations were measured in cecal contents. Cecal contents were processed by adding 0.1 N HCl and shaking (180×g) overnight at 25°C. Samples were vortexed and diluents collected and added to meta-phosphoric acid (HPO_3_, 25% w/v in distilled H_2_O), then centrifuged at 3000×g for 20 min. Supernatant was transferred into a gas chromatography vial (PerkinElmer) and analyzed by gas chromatography. Concentrations of SCFA were determined using external standards. Isocaproate was used as an internal standard.

#### Cytokine expression

After 35 days, mice were sacrificed and segments of small and large intestine were removed and snap-frozen at −80C. Cytokine expression was measured using a MesoScale Discovery Kit as per manufacturer’s instructions. IL-17 was measured by ELISA (R&D systems).

#### Microbial analysis

Stool were analyzed using terminal restriction fragment length polymorphism (T-RFLP). Total DNA was extracted using a FastDNA Spin Kit (MP Biomedical). 16S rRNA was amplified by PCR using a 6-FAM-5′-labelled, broad-range forward primer 6-FAM-8F (Applied Biosystems), 5′-AGAGTTTGATCCTGGCTCAG-3′) and a broad-range reverse primer 926R (Applied Biosystems) (5′-AGAAAGGAGGTGATCCAGCC-3′) [33]. Cycling conditions consisted of an initial denaturing step at 94°C for 2 min followed by 35 cycles of 94°C 1 min, 56°C 1 min, 72°C 1 min, and a final 10 min extension at 72°C. A DNA-free template control was included in every PCR run and amplification confirmed by visualization of a single 920 kb PCR product on a 1% agarose gel. Amplicons were purified using Qiagen MinElute PCR Purification Kit. Amplicon DNA (200–300 ng) was digested with the Hpall restriction enzyme (Promega, Madison, Wisconsin, USA) for 16 hours at 37°C. For each sample, 100 ng of HPAII digested fragments were resolved in duplicate using a 3130XL Genetic Analyzer (Applied Biosystems, Carlsbad, California, USA). Each sample was separated with an internal ROX1000 DNA marker to enable fragment length normalization. Bionumerics 6.0 software (Applied Maths, St-Martens-Latem, Belgium) was used to normalize fluorescently labeled terminal fragment lengths and select peaks of interest. Selected peaks of interest were associated, *in silico*, with fragment lengths of known bacteria using Microbial Community Analysis 3 and Ribosomal Database Project v.9 [34], [35].

#### Microbial data analysis

To reveal patterns in microbial composition between the groups, multivariate analysis was conducted using SIMCA-P+12.0 software (Umetrics, Sweden). Data was analyzed using both an unsupervised principle component analysis (PCA) and a supervised method in which class labels are known (partial least squares discriminant analysis (PLS-DA). PCA was used to reduce the dimensionality of the data while still retaining as much information as in the original data. This reduction is done by a linear transformation to a new set of variables (principle components) which are all uncorrelated to each other. Partial least squares (PLS) regression finds a linear regression model of two data sets, X, and Y using a series of local least square fits. PLS-DA is a special variant of the classic PLS only the Y are binary values, not continuous values. Partial least squares discriminant analysis (PLS-DA), a supervised pattern recognition approach, was used as a predictive model to identify differences in microbial composition.

### Statistical Analysis

All data are expressed as the mean ± SEM. One-way ANOVA followed by Dunnett’s multiple comparisons test was performed using GraphPad Prism version 6.00 for Windows, GraphPad Software, La Jolla California USA. Equality of variances was tested using the Bartlett’s test.

## Results

### Effects of Short Term PM_10_ Exposure on Gene Expression

#### Small intestine

Mice were gavaged daily with PM_10_ and expression of 96 genes in small intestinal tissue measured after 7 and 14 days. PM_10-_induced changes in gene expression differed between 7 and 14 days, suggesting host adaptation to continual exposure. Table 1 lists those genes that showed ≥1.5 fold change in expression as compared with baseline expression and Table S2 shows results for all 96 genes tested. After 7 days, genes up-regulated ≥1.5 fold in the small intestine included those involved in antigen presentation (*B2m*) and neutrophil, monocyte and T cell migration (*Sele, Cxcl10*) (Table 1). Genes down-regulated at 7 days in PM_10_ treated mice included cytokines (*Il13, Il12a, Il5, IFNg*), *Ccl19*, a chemokine involved in T cell trafficking to secondary lymph nodes, and *Cd19*, a cell marker for B cells. However, after 14 days of treatment, several genes which had originally shown a negative-fold change at 7 days now had a positive-fold change, including *Ccl19, Cd19, and Il12a*. This was associated with a down-regulation of *Il17, Sele, Il5, Il2, Agtr2*, and *Cd34*.

**Table 1:** Genes which showed changes of ≥1.5 fold in small intestine and colon at 7 and 14 days in response to PM_10_ treatment**.

Day	Tissue	Positive fold-change*	Negative-fold change*	Day	Tissue	Positive fold-change*	Negative fold-change*
**7**	**Small Intestine**	*Sele*	*Il13*	**14**	**Small Intestine**	*Cd19*	*Il7*
		*B2m*	*Cd19*			*Ccl19*	*Sele*
		*Cxcl10*	*Ccl19*			*Il12a*	*Il5*
			*Il12a*			*Cd40*	*Agtr2*
			*Il5*			*Ccr7*	*Il2*
			*Ifng*			*Ccl2*	*Cd34*
			*Agtr2*			*Il12b*	
			*Ccl3*				
			*Selp*				
			*Cd40lg*				
	**Colon**	*Ccr7*	*Cxcl11*		**Colon**	*Il12a*	*Il4*
		*Cd80*	*Il17*			*Il17*	*Il5*
		*Il12a*	*Csf2*			*Il2*	*Agtr2*
		*Sele*	*Tbx21*				*Tbx21*
		*Vcam1*	*Il4*				*Il1b*
		*Cd40lg*	*Ccl5*				*Ccl5*
		*Cd4*	*Il10*				*Il1a*
		*Cd86*	*Cd38*				*Ccl19*
		*Ccl19*	*Ece1*				
		*Il12b*	*Cd3e*				
		*Cd19*	*Cxcr3*				
		*Ifng*	*Socs1*				
		*Agtr2*	*Smad7*				
		*Icos*	*Tnf*				
		*Fn1*	*Nos2*				
		*Cd40*	*Cd8a*				
		*Ccl2*					
		*Cd68*					
		*Stat4*					
		*Tfrc*					
		*Cd28*					
		*Ptprc*					
		*Il2ra*					
		*Pgk1*					

* Only those genes showing ≥1.5 fold-change are included;

** Values and standard deviations of all genes tested are given in Supplementary Tables 2 and 3.

#### Colon

Table 1 lists those genes that showed ≥1.5 fold change in expression as compared with baseline expression and Table S3 shows results for all 96 genes tested. A similar difference in gene expression was also seen in the colon between 7 and 14 days (Table 1). After 7 days, 41 genes showed ≥1.5 fold change in expression. Genes up-regulated included those for pro-inflammatory cytokines (*Il12a, IL12b*, *IFNg*), monocyte and lymphocyte adhesion/migration molecules (*Sele, Vcam1, Fn1, Ccl2, Ccl19, Ccr7*), immune cell markers (*Cd4, Cd19*), and co-stimulatory receptors (*Cd80, Cd86, Cd40 and Cd40lg*). Genes down-regulated at 7 days in response to PM_10_ included cytokines (*Il4, Il10, Il17, Tnf*), cell signalling and signal transduction molecules (*Nos2, Socs1, Smad7*), transcription factors (*Tbx21*) and chemokines (*Ccl5, Cxcl11*). After 14 days, this response was significantly lessened, with only 11 genes showing altered expression. However, these included upregulation of genes for pro-inflammatory cytokines (*Il12a, Il17, Il2*) and a down-regulation of other cytokine genes (*Il4, Il5, Il1b, Il1a*).

### Ingenuity Pathway Analysis

**Figure 1:**
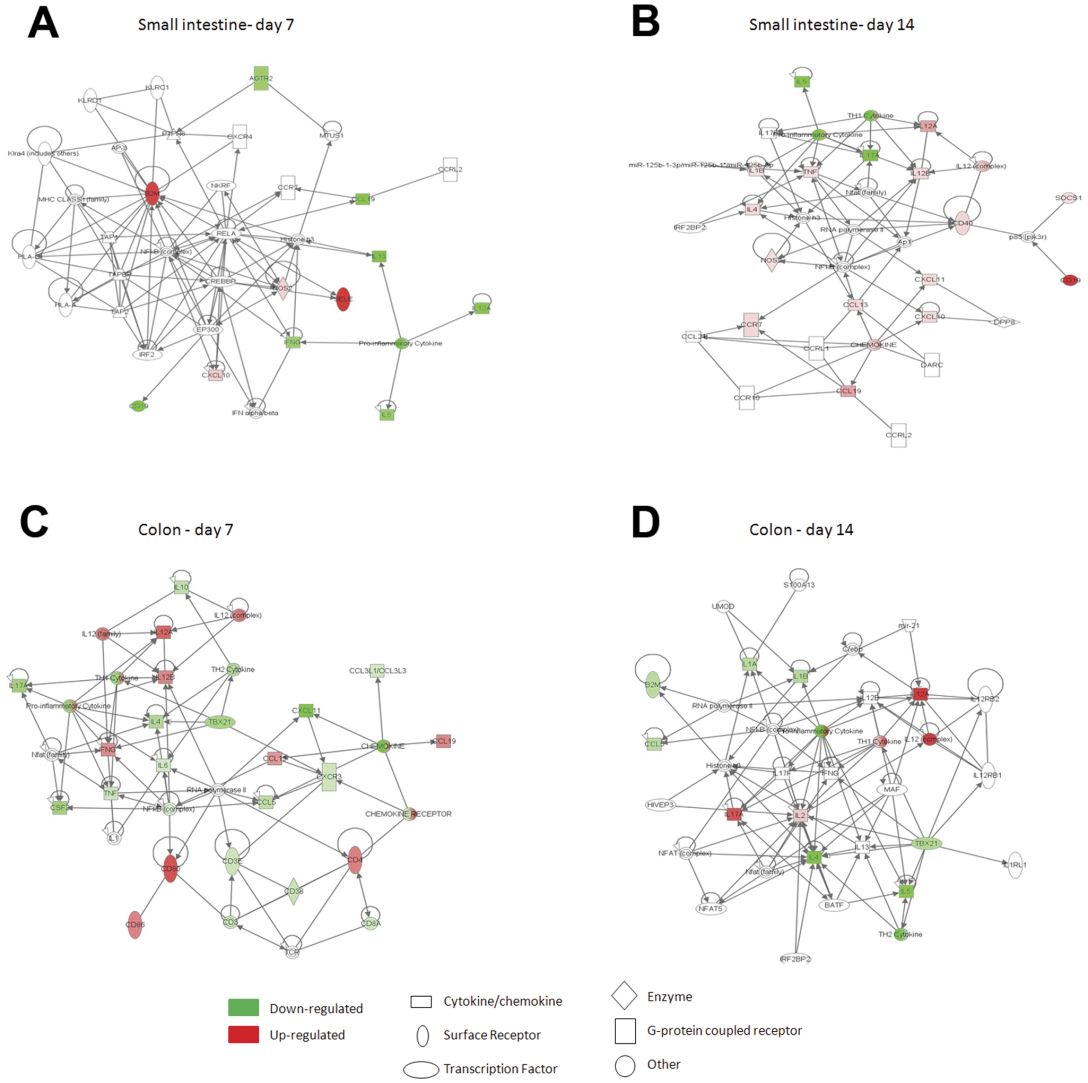
Ingenuity Pathway gene networks. The most highly significant gene networks identified in the Ingenuity Pathway analysis of the gene expression data in response to PM_10_ are shown for small intestine at days 7(A) and 14(B) and colon at days 7(C) and (D). The networks are displayed graphically as nodes (genes/gene products) and edges (the biological relationships between the nodes). The intensity of the node color indicates the degree of up (red) or down (green) regulation in gene expression. Nodes are displayed using shapes that represent the functional class of the gene product. Edges are displayed as a direct interaction (solid line).

Ingenuity Pathway Analysis (IPA) showed differences between small intestinal and colonic responses at 7 compared with 14 days. As seen in Figure 1A, PM_10_-induced changes in the small intestine at day 7 involved immune cell development and function. This was followed by an induction of pathways associated with immune cell signalling, interaction, and movement by day 14 (Figure 1B). In the colon at d7 (Figure 1C), a primary pathway involved NF-κB down-regulation with downstream effects including a downregulation of *Il6, Il4, Tnfa, Csf*, and *Il17a*, and an upregulation of *Cd4, Cd8, Ifng* and the co-stimulatory molecules *Cd80* and *Cd86* was seen. By day 14 (Figure 1D), an upregulation of *Il17a, Il12*, and *Il2* was seen along with a downregulation of *Il4* and *Il5*.

### PM_10_ Exposure Elicits a Transient Increase in Pro-cytokine Secretion in the Small Intestine

**Figure 2:**
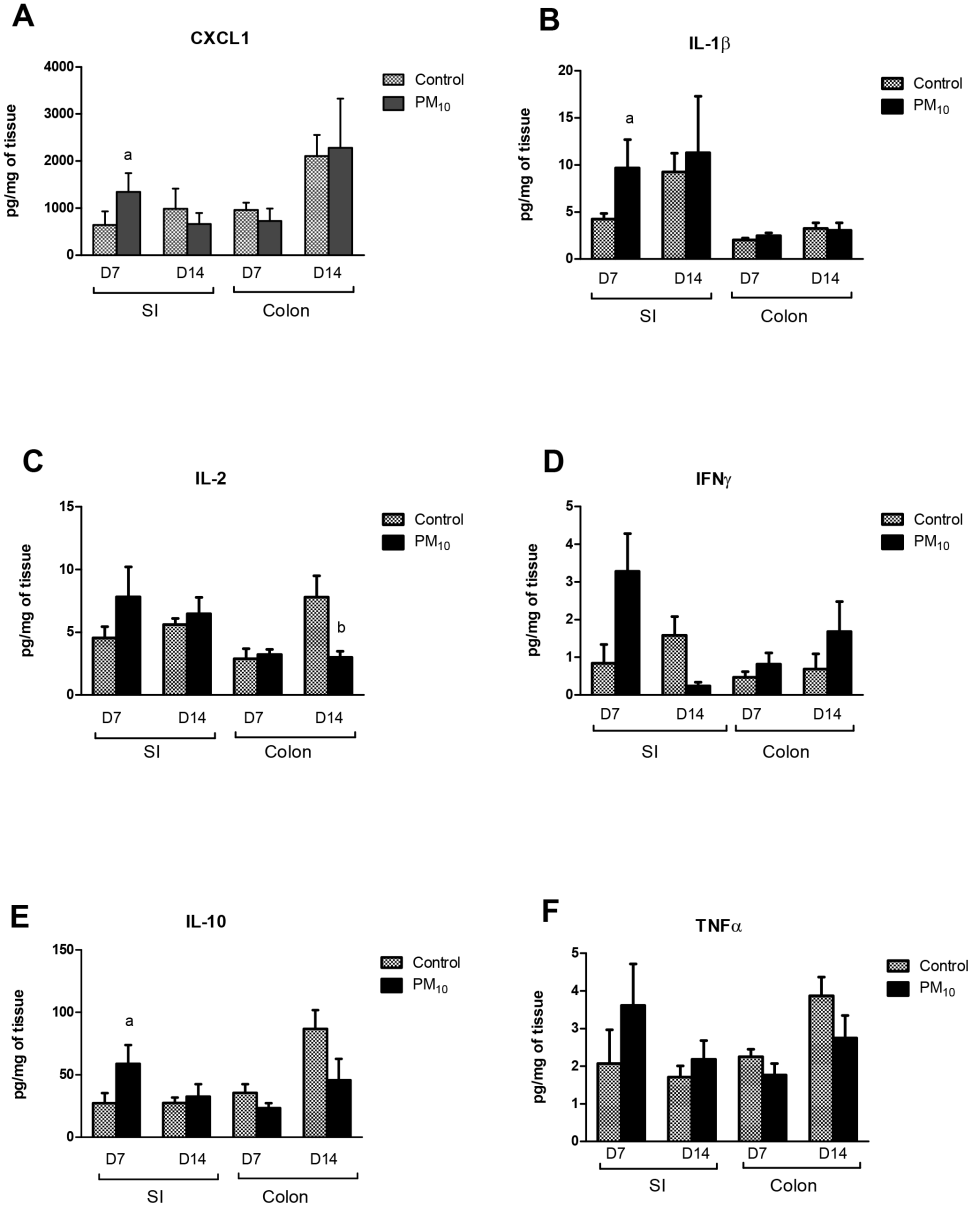
Cytokine secretion from isolated small intestine (SI) and colon taken from WT mice treated with PM_10_ for 7 (D7) or 14 (D14) days. A significant increase in CXCL1 (A), IL-1β (B) and IL-10 (E) was seen in the small intestine at day 7. Bars are mean ± SEM n = 6 for all groups ^a^ p<0.05 compared with control at day 7; ^b^p<0.05 compared with control at day 14.

To examine if the alteration in gene expression was accompanied by changes in basal cytokine secretion, segments of intestine were cultured and cytokine secretion measured. A significant increase in basal secretion of CXCL1, IL-1β, and IL-10 was seen in the small intestine of mice exposed to PM_10_ for 7 days (Figure 2). However, after 14 days of treatment, despite changes in gene expression, in the small intestine only a decrease in IFNγ secretion was seen in mice treated with PM_10_ while in the colon, a decrease in basal IL-2 secretion was observed (Figure 2).

### PM_10_ Increases Gut Permeability and Hyporesponsiveness in Splenocytes

**Figure 3:**
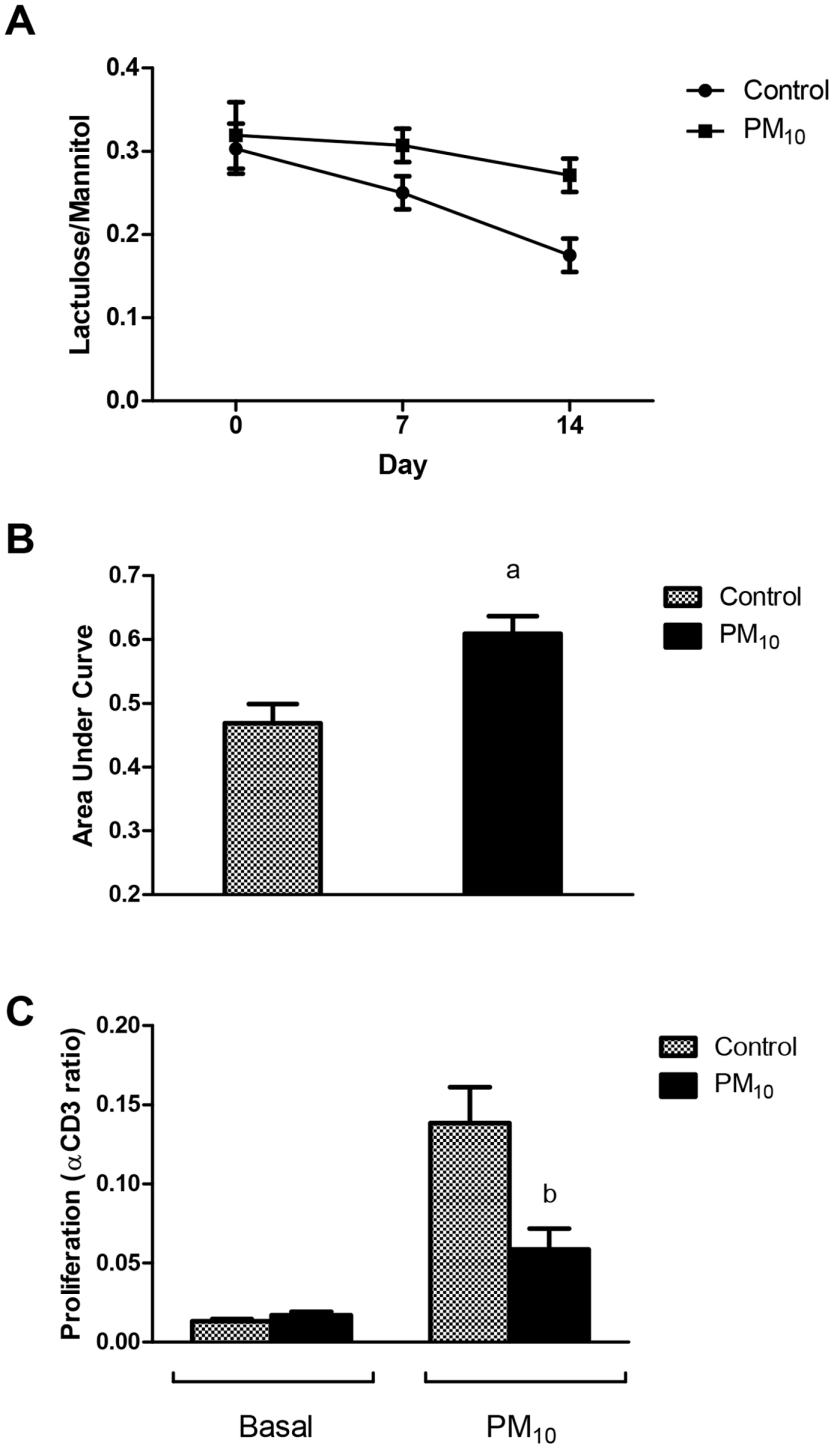
PM_10_ increased small intestinal permeability and induced hyporesponsiveness in splenocytes. A) Lactulose/mannitol excretion in urine was measured weekly. B) Statistical analysis of areas under the curve of mice treated with PM_10_ or vehicle. Small intestinal permeability from the PM_10_ treated group was significantly higher compared with vehicle treated group. C) Proliferation of isolated splenocytes from mice treated for 14 days under basal conditions and in response to PM_10_ (0.5 mg/ml). Response to αCD3 was used as a positive control and results are displayed as a ratio to αCD3 proliferation. Splenocytes isolated from mice that had been treated with PM_10_ for 14 days had less proliferation in response to PM_10_ compared with control mice. ^a^p<0.05 compared with control mice, n = 8; ^b^p<0.05 compared with control in response to PM_10._ N = 6.

Mice receiving PM_10_ exhibited increased gut permeability over the 14-day period compared with control mice as evidenced by an increase in the lactulose/mannitol ratio (Figure 3A). Analysis of the area under the curve revealed a significant increase in PM_10_ treated mice (Figure 3B). This was associated with a decreased proliferation in isolated splenocytes in response to PM_10_ in mice which had been treated with PM_10_ for 14 days (Figure 3C), suggesting that continual exposure of splenocytes to PM_10_ over the 14 days resulted in a decreased response to the particulate matter. There was no difference in basal proliferation between groups.

### Effects of Long-term Exposure on Disease Parameters

**Figure 4:**
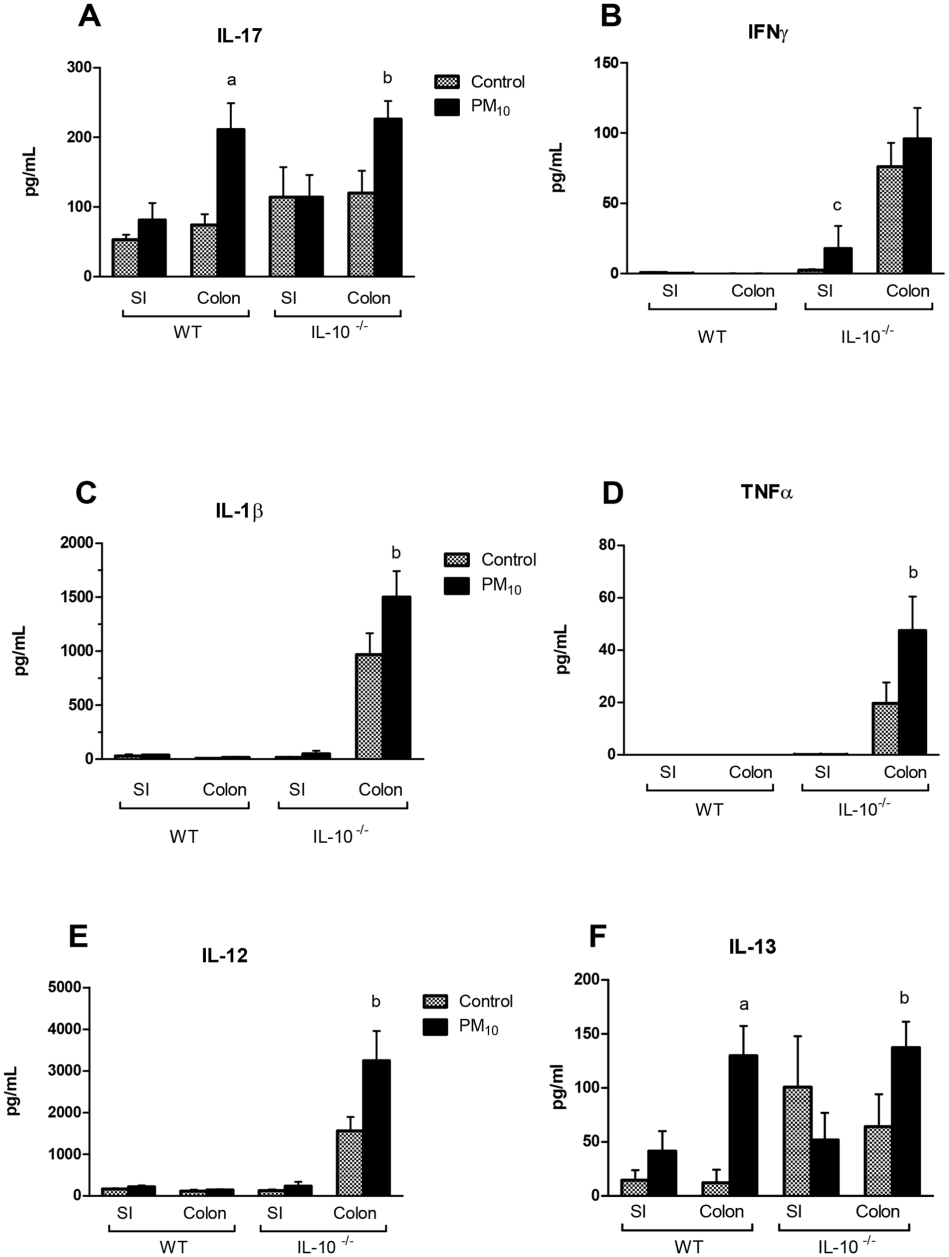
Cytokine expression in small intestine (SI) and colon taken from WT and IL-10^−/−^ mice treated with PM_10_ for 35 days. A significant increase in IL-17 (A), IL-1β (c), TNFα (D), IL-12 (E) and IL-13 (F) was seen in the colon of IL-10^−/−^ mice treated with PM_10_. PM_10_ induced increased IL-17 and IL-13 in the colons of WT mice. Bars are mean ± SEM n = 6–9 for all groups ^a^ p<0.05 compared with WT control colon; ^b^p<0.05 compared with IL-10^−/−^ control colon; ^c^p<0.05 compared with IL-10^−/−^ control SI.

Having shown transient alterations in intestinal gene expression and increases in gut permeability following a short-term treatment with PM_10_, the next series of experiments were performed to examine effects of a longer exposure (35 days), and also to determine if a transient increase in gut permeability would exacerbate colitis in the IL-10^−/−^ mouse. Following 35 days of exposure to PM_10_, 3 out of 8 WT mice had an increased mononuclear and/or neutrophilic infiltration into the lamina propria in the colon (Table 2). In the IL-10^−/−^ mice, an increased severity of histological damage was observed as evidenced by increased enterocyte injury, epithelial hyperplasia, and lamina propria neutrophil infiltrate (Table 2). There was no significant injury in the small intestine in any of the groups. There was no difference in weight gain between the groups (data not shown). This increase in histological evidence of disease was accompanied by an enhanced expression of colonic pro-inflammatory cytokines, IL-17 and IL-13, in the WT mice, and increased expression of the pro-inflammatory cytokines IL-17, IL-1β, TNFα, IL-12, and IL-13 in the IL-10^−/−^ mice (Figure 4A–F). In addition, IL-10^−/−^ mice also showed increased expression of the pro-inflammatory cytokine, IFNγ in the small intestine. These data indicate a a worsening of disease in the presence of particulate matter in the IL-10^−/−^ mice.

**Table 2:** Histological scores for WT and IL-10^−/−^ mice after treatment with PM_10_ for 35 days.

			Histological Score					
Strain	Tissue	Group	# of Affected Mice	Enterocyte Injury (0–3)	Epithelial hyperplasia(0–3)	Mononuclear Infiltrate(0–3)	Neutrophilic Infiltrate(0–2)	Total Score (0–10)
WT	SI	Control	0/7	0	0	0	0	0
		PM_10_	1/8	0	0	1	0	1
	Colon	Control	0/7	0	0	0	0	0
		PM_10_	3/8	0	0	0.67±0.38	1.0±0.58	1.7±0.9
IL-10^−/−^	SI	Control	3/9	0	0	0.28±0.18	0	0.28±0.18
		PM_10_	0/7	0	0	0	0	0
	Colon	Control	8/9	0.7±0.3	0	1.0±0.2	0.6±0.2	2.2±0.6
		PM_10_	7/7	1.5±0.6	0.17±0.06	1.5±0.6	1.5±0.6	4.7±1.4*

* p<0.05 compared compared with colons from WT control, WT PM_10_, and IL-10^−/−^ control.

### Long Term PM_10_ Exposure Alters Microbiome

**Figure 5:**
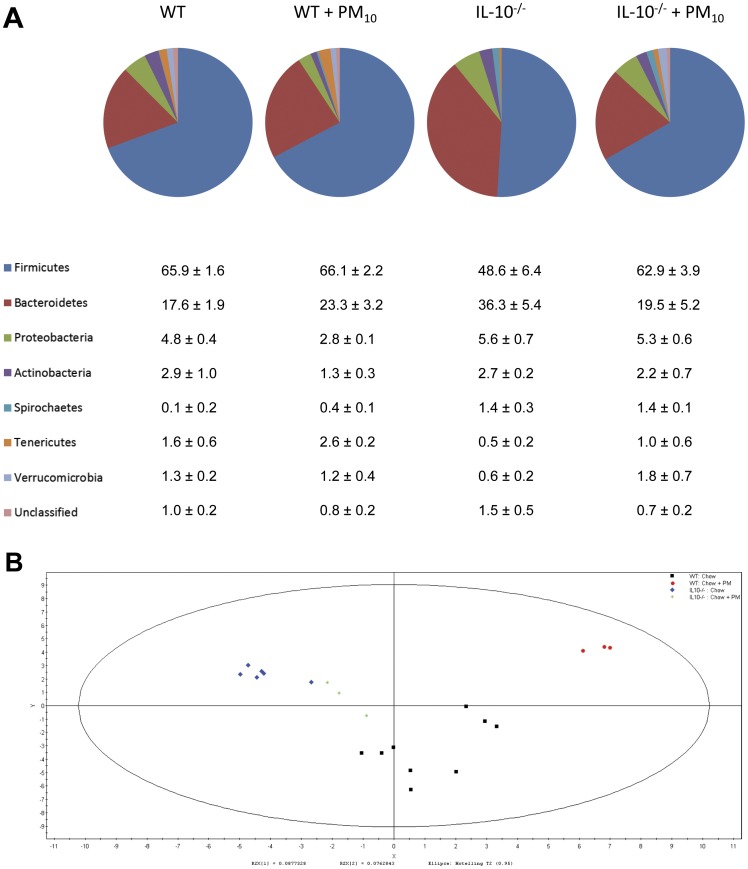
Microbiota composition in stool samples from WT and IL-10^−/−^ mice after 35 days of treatment with PM_10_. Stool was analyzed using T-RFLP. (A) Relative abundance of phyla. Table provides mean ± SEM for each phylum as the percentage of total sequences. (B) Bacteria communities were clustered using partial least squares discriminant analysis (PLS-DA). WT mice clustered independently from IL-10 mice, and PM_10_ treatment shifted the microbiota in both WT and IL-10^−/−^ groups. WT control: Black squares (n = 9); IL-10^−/−^ control: Blue triangles (n = 6); WT+PM_10_: Red circles (n = 3); IL-10^−/−^+PM_10_: green crosses (n = 3).

**Figure 6:**
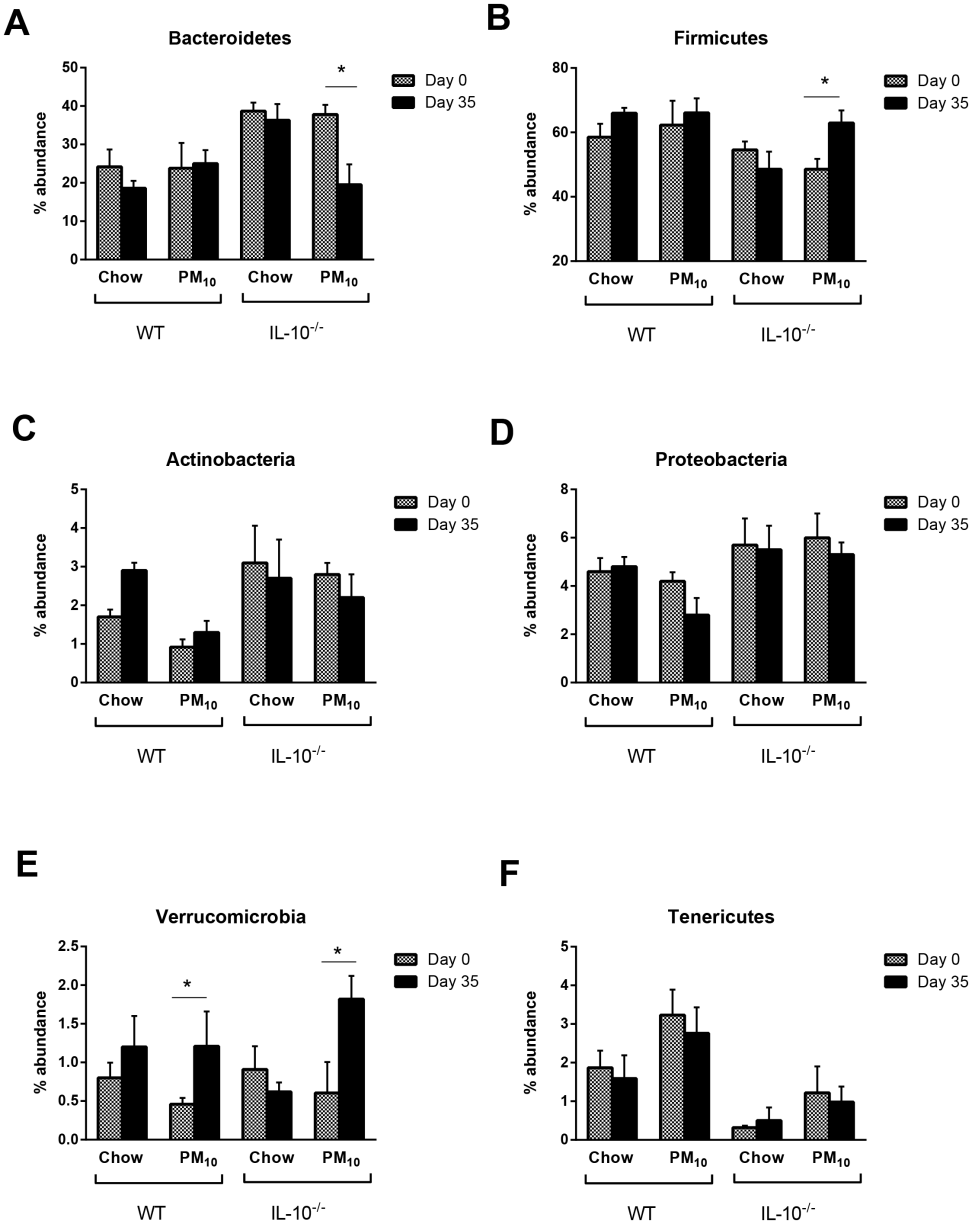
Relative abundance of phyla in stool samples from WT and IL-10^−/−^ mice at days 0 and 35. In IL-10^−/−^ mice, PM_10_ decreased percentages of Bacteroidetes and increased Firmicutes compared with day 0 (Figure 6). PM_10_ increased amounts of Verrucomicrobia in both WT and IL-10^−/−^ mice. Bars are mean ± SEM n = 6–9 for all groups ^*^p<0.05 compared with day 0.

Colitis in the IL-10^−/−^ mouse is dependent upon the gut microflora, and treatments aimed at changing the gut microflora can significantly attenuate or exacerbate disease [24], [32]. To determine if the alterations in disease and cytokine secretion were associated with changes in the gut microflora, stool samples were analyzed. Analysis of the microbial composition of stool samples by T-RFLP showed differences in relative abundance of phyla in both IL-10^−/−^ mice compared with WT, and in PM_10_ treated mice (Figure 5A). Multivariate analysis (PLS-DA; Figure 5B) demonstrated that WT and IL-10^−/−^ mice clustered separately and apart from the groups receiving PM_10_. In order to determine if there was a time and age-related effect on gut microbiota, the relative abundance of gut microbiota was compared between samples taken at day 0 and samples taken at day 35. In Figure 6, it can be seen that in WT and IL-10^−/−^ mice on chow, there were no significant changes in relative abundance of Bacteroidetes, Firmicutes, Actinobacteria, Proteobacteria or Verrucomicrobia over 35 days, indicating no specific age-related effects. The addition of PM_10_ to the chow increased amounts of Verrucomicrobia in both WT and IL-10^−/−^ mice, and decreased percentages of Bacteroidetes and increased percentages of Firmicutes in IL-10*^−/−^* mice compared with day 0 (Figure 6).

### Short Chain Fatty Acid (SCFA) Composition

**Figure 7:**
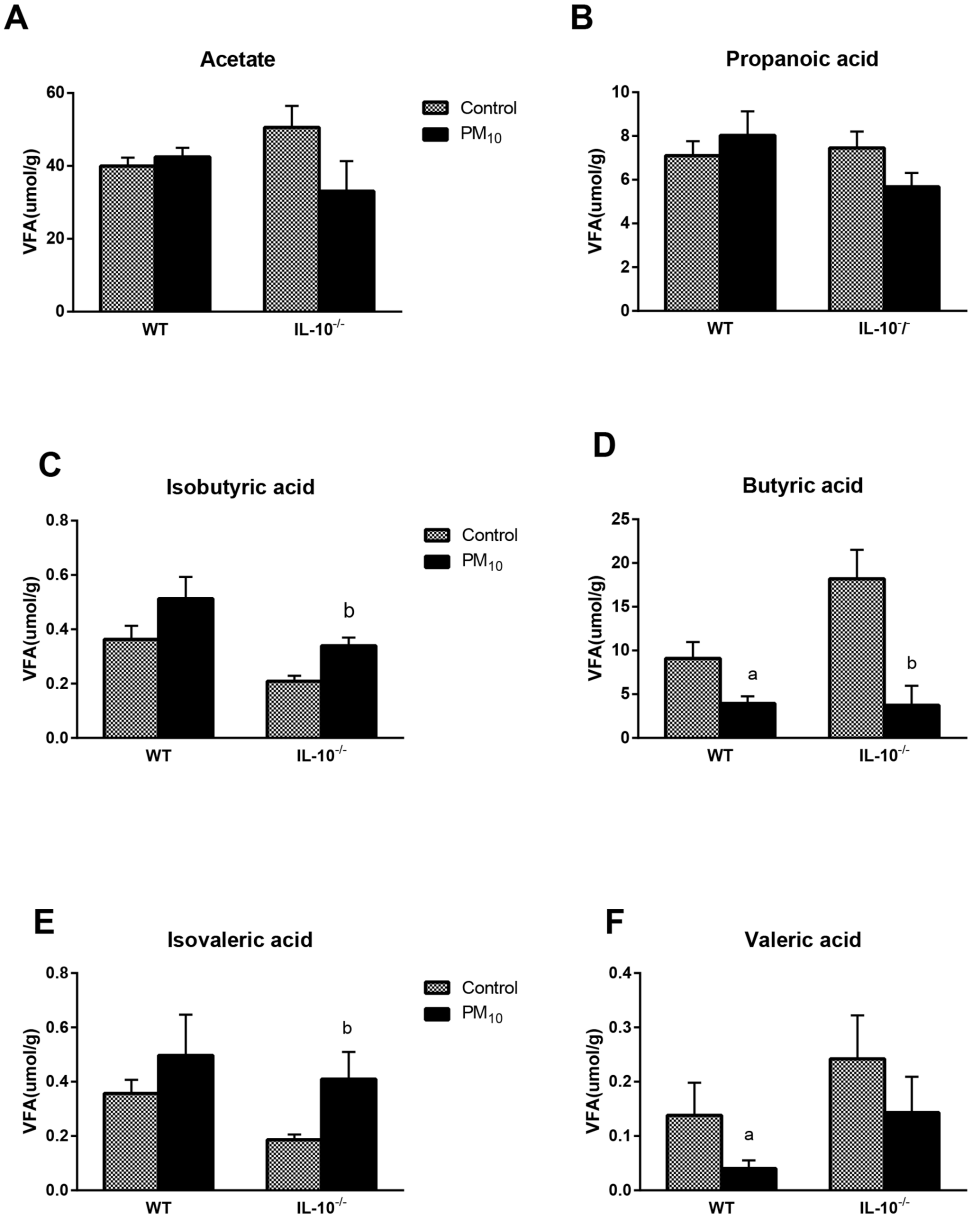
Short chain fatty acids in cecal contents. IL-10^−/−^ mice exposed to PM_10_ showed a significant increase in isovaleric (F) and isobutyric (C) and a decrease in butyric acid (D). WT mice had a significant decrease in butyric acid (D) and valeric acid (F). Bars are mean ± SEM n = 6 for all groups ^a^ p<0.05 compared with WT control mice; ^b^p<0.05 compared with IL-10^−/−^ control mice.

SCFA, in particular butyrate and acetate, have significant effects on intestinal immune and barrier function [36]. IL-10^−/−^ mice exposed to PM_10_, showed a significant increase in the cecal concentration of the branched fatty acids isovalerate and isobutyrate (Figure 7C and E), along with a decrease in butyrate (Figure 7D). WT mice had a significant decrease in butyrate and valerate (Figure 7D and F).

## Discussion

In this study we demonstrate that short-term exposure of the gut to high levels of airborne particulate matter results in an increased gut permeability and heightened innate immune response in the small intestine while chronic exposure results in enhanced expression of pro-inflammatory cytokines and alterations in microbiome composition and function in the colon. In addition, long-term exposure exacerbated colitis in the IL-10^−/−^ mouse model.

Associations have been shown to exist between air pollution exposure and an increased risk of appendicitis [12], abdominal pain [37], and hospitalizations in patients with inflammatory bowel disease (IBD) [15], [38]. Findings from this study that acute PM_10_ exposure resulted in increased expression of genes related to chemotactic activity of monocytes and activated T cells along with an increase in the secretion of CXCL1 and IL-1β suggests that an enhanced secretion of pro-inflammatory cytokines induced by particulate matter could contribute to the abdominal pain associated with air pollution exposure. The overall response of the small intestine suggested an acute innate immune response with stimulation of inflammasome activity, similar to what has been reported in lung tissue exposed to particulate matter [39], [40], [41].

Both gene expression and basal cytokine secretion differed between the 7 and 14 day treatment groups. In particular, by 14 days a PM_10_-induced increase in basal secretion of cytokines was no longer evident, suggesting that the initial innate immune response had been dampened by continual exposure. Gene expression had also changed, as several genes that were up-regulated at 7 days were down-regulated at 14 days (*Cd19, ccl19, Il12a*). Interestingly, the increase in *cd19*, a receptor on B cells that lowers the threshold needed for activation, suggested a possible priming of the adaptive immune system along with a shift towards a humoral immune response. This reduced small intestinal tissue response to continual exposure may be related to a particle-induced hyporesponsiveness. The finding that splenocytes from mice which had been treated with PM_10_ had a reduced proliferation when incubated directly with the particles, supports this hypothesis. This suppression by PM_10_ exposure is similar to the hyporesponsiveness that diesel exhaust particles (DEP) induce in human peripheral blood monocytes [42]. Of interest is the finding that this suppressive effect of DEP was shown to result in an increased survival of *Mycobacterium tuberculosis* in DEP-exposed macrophages [42] and also to an increased pulmonary *M. tuberculosis* load in mice due to a decreased clearance of microbes [43]. The gastrointestinal tract is densely colonized by a complex microbiota comprised of >1000 microbial species [44]. Thus, a similar effect in the gut may allow for an increased number of microbes to come into contact with the gut epithelium, which could both initiate and perpetuate existing gut inflammatory responses. Indeed, patients with inflammatory bowel disease exhibit increased numbers of microbes associated with the gut mucosa [45]–[49], and innate immune defects have been shown to exist in patients with Crohn’s disease [50].

Interestingly, the colon appeared to respond differently than the small intestine to the particulate matter, which may be related to either the concentration of particulate matter which reached the colon, or alternatively, a longer period of exposure in the colon due to decreased motility. A much larger number of genes showed an up-regulation at 7 days of PM_10_ treatment, as compared with the small intestinal response. After 7 days of PM_10_ exposure, a significant decrease in the expression of Csf2 (a growth factor needed for the production, differentiation, and function of granulocytes and macrophages), *Il10, tnf, Socs1*, *Smad7*, and *Nos2* gene expression was observed, which indicates a possible decrease in macrophage production and effector function in colonic tissue. There was also a significant increase in *Cd86* (B7.2), and *Cd4* gene expression, indicating a stimulation of T cell activation and proliferation. However, as was seen in the small intestine, this stimulation of innate immune response was not evident by 14 days, and indeed, by 14 days, had been replaced with an up-regulation of *Il12a, Il17*, and *Il2*, all indicative of a heightened adaptive immune response. The increase in gene expression of *Il12a, Il17, and Il2* was not accompanied by an increase in basal secretion of these cytokines in the colon. However, we did not measure tissue protein expression or protein secretion in response to any stimulus, so it is possible that there were increased protein levels within the epithelial tissue that could result in a hyper-response to a stimulatory condition. Further experiments are required to determine how the presence of particulate matter would affect the gut response to a stimulus such as an infectious organism.

Increases in gut permeability have been associated with numerous diseases, including inflammatory bowel disease, diabetes, and celiac disease^,^ [20], [51]. The initial inflammatory response induced by PM_10_ in the small intestine was accompanied by increased permeability. This may have occurred as a direct result of PM_10_ on epithelial cells, or alternatively, as a secondary effect due to PM_10_ effects on immune cell function [52]. PM_10_ has been shown to generate oxygen free radicals which induce oxidative stress in the epithelia causing increased permeability due to disruptions in tight junctions [20]. Long term exposure of WT and IL-10^−/−^ mice to PM_10_ resulted in significant alterations in cytokine expression within the colon. While WT mice had enhanced IL-17 and IL-13 expression in the colon, IL-10^−/−^ mice had increased expression of IL-17, IL-1β, TNFα, IL-12, and IL-13. This enhanced pro-inflammatory cytokine expression was associated with an exacerbation of disease in the IL-10^−/−^ mice as evidenced by an increased histological score. Elevated levels of IL-17 have been shown to be associated with numerous autoimmune and inflammatory diseases and to be linked with the presence of specific strains of microbes [53], [54]. This enhanced response in the IL-10^−/−^ mouse suggests that under conditions of genetic susceptibility, an exposure to particulate matter could trigger and accelerate the development of inflammatory disease through an increase in gut permeability and decreased ability to handle gut microbes. These findings may have clinical relevance, in that patients with existing low grade inflammation may react much more to particulate matter compared with healthy individuals.

In the IL-10^−/−^ mouse, cytokine expression and inflammation depends upon the presence of microbiota; mice housed in germ free environments do not develop colitis and treatments aimed at modifying gut bacteria can both prevent and treat the colitis [23], [32], [55]. The IL-17-secreting CD4+ T cell subset has been shown to be induced by specific microbes within the gut [53], [54], [56]. For these studies, we employed terminal restriction fragment length polymorphism (T-RFLP) technology as a rapid and reproducible method to assess microbial patterns over time in the different groups of mice [57]. While the community patterns generated by T-RFLP are consistent with other DNA fingerprinting techniques and are useful for estimating the phylogenetic diversity and composition of complex microbial communities, this method may underestimate bacterial diversity due to inherent biases associated with this method, including those that arise from sample collection, DNA extraction, and the fact that it is PCR-based [33]. In addition, matching T-RFs to databases or clone libraries can also be biased by the choice of restrictive enzymes and fluorescent dyes [58]. Thus, the phylogenetic composition of samples must be considered to be semiquantitative. However, this technique is valid for comparing communities over time and for comparing microbial communities between different treatment groups.

Multivariate analysis revealed a PM_10_-induced shift in microbial composition in both wild-type and IL-10^−/−^ mice. This was evidenced both by changes in the relative proportions of microbes as well as changes in SCFA. IL-10^−/−^ mice demonstrated a significant increase in concentrations of the branched chain fatty acids isobutyrate and isovalerate. Isovalerate is a known inhibitor of succinyl-CoA ligase in the tricarboxylic acid cycle and has been shown to inhibit mitochondrial oxygen consumption [36], [59]. In addition, of particular note was the decrease in butyrate concentration, which is important in colonic epithelial cell metabolism and in the induction of host defense peptides [36], [60], [61]. Depletion of butyrate has been shown to result in decreased barrier function and increased susceptibility to inflammation [36]. Isobutyrate and isovalerate originate from conversion of the amino acids isoleucine, leucine, or valine via the Stickland reaction. Amino acid fermentation via the Stickland reaction is carried out by proteolytic clostridia and is indicative of a shift from carbohydrate fermentation to protein fermentation. Overall, these results suggest that exposure of the gut to PM_10_ significantly affects both the composition and function of the colonic microbiome and possibly induces the development of a more inflammatory luminal environment which contributes to the induction of pro-inflammatory cytokines in the host. Whether these alterations in gut microbiota were caused by PM_10_ directly, occurred due to PM_10_-induced immune changes or a combination of both is unknown, and requires further study.

PM_10_ is a highly complex mixture of elemental and organic carbon, metals, sulfates, nitrates, and organic contaminants [3], [62], [63], [64]. Numerous studies have demonstrated effects of metals, including iron, vanadium, and copper on host physiology due to their redox potential [65], [66], [67], while others have demonstrated inflammatory responses to the soluble fraction of the particulate matter [63]. However, Fujii et al [6] demonstrated that the soluble compounds from the EHC-93 mixture had a smaller effect on cytokine production by bronchial epithelial cells compared with the particles themselves. The PM_10_ material contains small amounts of endotoxin (∼3ng/100 µg of particles) as measured by the Limulus Amebocyte Lysate Test [6], [68]. However, previous studies have examined the role of endotoxin in the PM-induced effects seen in lung, and have concluded that endotoxin contamination cannot explain either cytokine production by bronchial epithelial cells exposed to EHC-93 or the bone marrow stimulation induced by EHC-93 particles ( [6], [68]. Thus, it is unlikely that the effects seen due to oral ingestion are due to the minor amounts of endotoxin present, especially considering the large amount of endotoxin normally present in the gut. In addition, particulate matter will be acted upon by digestive enzymes and gastric acid; thus changing the chemical nature of the material as it passes along the intestinal tract.

These studies were designed to mimic a continual exposure of an individual to contaminated food and high levels of particulate matter. However, this has to be considered a limitation of this study, as the concentration of particulate matter the gut may be exposed to is unknown. Levels of PM in urban environments can range anywhere from 20 to 1000 µg/m^3^ at peak concentrations resulting in a total inhaled dose over 24 hours of up to 20,000 µg [28]. Depending upon their size, these particles are deposited throughout the lung, with larger particles primarily deposited within the oropharynx region. These particles can be retained for an indeterminate amount of time; for instance, a study by Semmler-Benke [69] demonstrated that iridium labelled carbon black nanoparticles given via inhalation to rats could be detected in the feces up to 6 months following administration. Along with inhalation, dietary intake of contaminated food and drinking water can also occur, which would compound exposure [18]. It has been estimated that 10^12^–10^14^ particles are ingested per individual per day from the typical western diet with an estimated mucosal uptake of ∼1% (10^9^–10^12^/day) [70]. Some of these particles are added to food items, and previous studies have shown these particles to accumulate over time in intestinal macrophages [71]. A study by Lomer et al [72] calculated a median particulate intake of 35 mg/individual/day for aluminosilicates. Another study estimated that average daily intakes of nickel from contaminated food can range from 70–660 µg/day [19]. This would compare with ∼25 µg/day of nickel that the mice in our study would have received. Our findings that chronic exposure results in changes within the colon related to immune function and microbial colonization rather than in the small intestine suggest that a longer time of exposure in the colon may indeed occur and lead to these changes. The doses of PM_10_ used in these experiments are higher than what an individual would be exposed to via inhalation alone and are similar to those used by Mutlu et al [20]. In their studies, it was calculated that a dose of 200 µg of PM would equal ∼25 mg of human PM exposure; this would be ∼ 7 times higher than what would occur in a normal individual by inhalation alone. Our studies used a similar concentration (270 µg/day). Further studies using lesser doses of PM are required to determine if PM_10_ at lower levels would elicit similar responses.

In conclusion, our data demonstrate that exposure of the gut to particulate matter associated with air pollution initiates inflammatory responses within the small and large intestine and alters the colonic microbiome. These effects could have significant implications for patients with pre-existing disease, and provide a mechanism by which exposure to airborne particulate matter can trigger the onset of gastrointestinal disease.

## Supporting Information

Table S1 **PAH, Ion, and Metal composition of PM_10_ (EHC-93).**
(PDF)

Table S2 **Effects of 7 and 14 day treatment with PM_10_ on gene expression in small intestine in wild-type mice.**
(PDF)

Table S3 **Effects of 7 and 14 day treatment with PM_10_ on gene expression in colon in wild-type mice.**
(PDF)
