# Supplementary material for: Environmental Particulate Matter Induces Murine Intestinal Inflammatory Responses and Alters the Gut Microbiome
Source: PLoS One. 2013 Apr 24;8(4):e62220. doi: 10.1371/journal.pone.0062220 (PMC3634745; doi:10.1371/journal.pone.0062220)
Supplement: Table S1 — PAH, Ion, and Metal composition of PM10 (EHC-93). (PDF) [file pone.0062220.s001.pdf]

Table S1: PAH, Ion, and Metal composition of PM_{10} (EHC-93)

PAH composition	µg of PAH/g particle
Acenaphtene	0.20
Anthracene	0.54
Benzo[a]anthracene	1.10
Benzo[b]fluoranthene	2.78
Benzo[ghi]perylene	1.52
Benzo[a]pyrene	0.95
Benzo[e]pyrene	1.09
Chrysene	1.66
Indeno[1,2,3cd)pyrene	1.19
Fluoranthrene	2.47
Phenanthrene	1.83
Perylene	0.28
Pyrene	2.11
**Ion composition**	**µg of ions/g particle**
Sulfate ion	45 x 103
**Metal composition**	**µg of metals/g particle (% solubility in water)**
Aluminum	10 x 103 (2%)
Chromium	42 (3%)
Copper	845 (17%)
Iron	15 x 103 (1%)
Lead	7 x 103 (4%)
Magnesium	7 x 103 (14%)
Nickel	67 (7%)
Vanadium	90 (0%)
Zinc	10 x 103 (46%)

Table adapted from Vincent et al [1, 2].
Data is expressed in ug/g of particulate material.
