# Supplementary material for: Environmental Particulate Matter Induces Murine Intestinal Inflammatory Responses and Alters the Gut Microbiome
Source: PLoS One. 2013 Apr 24;8(4):e62220. doi: 10.1371/journal.pone.0062220 (PMC3634745; doi:10.1371/journal.pone.0062220)
Supplement: Table S2 — Effects of 7 and 14 day treatment with PM10 on gene expression in small intestine in wild-type mice. (PDF) [file pone.0062220.s002.pdf]

Table S2. Effects of 7 and 14 day treatment with PM_{10} on gene expression in small intestine in wild-type mice.

Class	Gene	Log_2(Fold Change)		Standard Deviation
Apoptosis	*Bax*	7 Day	-0.33	0.23
		14 Day	-0.10	0.24
	Bcl2	7 Day	0.08	0.36
		14 Day	-0.28	0.41
	Bcl2l1	7 Day	-0.21	0.30
		14 Day	-0.32	0.20
	Fas	7 Day	-0.15	0.36
		14 Day	0.17	0.22
	Fasl	7 Day	-0.31	0.81
		14 Day	-0.31	0.46
Cellular Marker	*B2m*	7 Day	2.67	0.20
		14 Day	-0.17	0.19
	Cd19	7 Day	-1.45	2.61
		14 Day	3.07	2.01
	Cd28	7 Day	-0.35	1.21
		14 Day	-0.29	0.98
	Cd34	7 Day	-0.26	0.42
		14 Day	-0.59	0.39
	Cd38	7 Day	-0.14	0.43
		14 Day	-0.40	0.22
	Cd3e	7 Day	-0.39	1.01
		14 Day	-0.07	0.60
	Cd4	7 Day	-0.14	0.62
		14 Day	0.15	0.56
	Cd40	7 Day	-0.10	0.64
		14 Day	0.69	0.68
	Cd40lg	7 Day	-0.60	1.89
		14 Day	0.14	1.25
	Cd68	7 Day	-0.21	0.38
		14 Day	-0.27	0.29
	Cd80	7 Day	0.17	0.61
		14 Day	-0.15	0.26
	Cd86	7 Day	0.12	0.48
		14 Day	0.00	0.29
	Cd8a	7 Day	-0.30	0.67
		14 Day	0.03	0.47
	Ctla4	7 Day	-0.47	0.71
		14 Day	0.20	0.57
	H2-Eb1	7 Day	0.01	0.93
		14 Day	-0.20	0.70
	Il2ra	7 Day	0.09	0.42

		14 Day	-0.52	0.38
	Ptprc	7 Day	-0.48	0.24
		14 Day	-0.13	0.25
	Ski	7 Day	-0.08	0.31
		7 Day	-0.27	0.45
	Tbx21	14 Day	-0.04	0.99
		7 Day	-0.03	0.85
	Tnfrsf18	14 Day	-0.35	0.49
		7 Day	-0.02	0.40
Cellular Migration	*Col4a5*	14 Day	0.07	0.28
		7 Day	0.11	0.41
	Fn1	14 Day	-0.22	0.26
		7 Day	-0.18	0.28
	Lrp2	14 Day	-0.22	0.81
		7 Day	-0.50	0.28
	Sele	14 Day	2.77	2.04
		7 Day	-1.26	1.94
	Selp	14 Day	-0.73	0.47
		7 Day	-0.17	0.47
	Vcam1	14 Day	-0.33	0.63
		7 Day	-0.02	0.23
Chemokine	*Ccl19*	14 Day	-1.29	2.30
		7 Day	1.89	2.02
	Ccl2	14 Day	0.37	0.50
		7 Day	0.61	0.58
	Ccl3	14 Day	-0.76	0.81
		7 Day	0.04	0.64
	Ccl5	14 Day	-0.34	1.19
		7 Day	-0.34	0.75
	Ccr2	14 Day	-0.20	0.49
		7 Day	-0.24	0.20
	Ccr4	14 Day	-0.19	1.20
		7 Day	-0.07	0.55
	Ccr7	14 Day	-0.33	1.33
		7 Day	0.66	1.41
	Cxcl10	14 Day	0.67	1.06
		7 Day	0.58	1.17
	Cxcl11	14 Day	-0.15	0.78
		7 Day	0.45	0.79
	Cxcr3	14 Day	-0.03	1.02
		7 Day	0.05	0.76
Cytokine	*Csf1*	14 Day	0.09	0.61
		7 Day	-0.20	0.45
	Csf2	14 Day	-0.10	0.48
		7 Day	-0.15	0.59
	Ifng	14 Day	-0.85	3.18
		7 Day	-0.51	2.70

	Il10	14 Day	-0.42	0.53
		7 Day	-0.31	0.44
	Il12a	14 Day	-1.27	1.99
		7 Day	1.84	1.68
	Il12b	14 Day	-0.03	0.51
		7 Day	0.61	0.62
	Il13	14 Day	-1.54	2.59
		7 Day	-0.29	0.72
	Il15	14 Day	-0.43	0.25
		7 Day	-0.31	0.17
	Il17	14 Day	0.33	2.22
		7 Day	-1.33	2.82
	Il18	14 Day	0.04	0.40
		7 Day	-0.02	0.42
	Il1a	14 Day	-0.56	0.30
		7 Day	-0.12	0.45
	Il1b	14 Day	-0.09	0.43
		7 Day	0.28	0.10
	Il2	14 Day	-0.58	1.06
		7 Day	-0.66	0.37
	Il4	14 Day	-0.22	1.65
		7 Day	0.58	3.57
	Il5	14 Day	-0.96	0.76
		7 Day	-1.09	1.84
	Il6	14 Day	-0.46	1.38
		7 Day	-0.05	0.79
	Il7	14 Day	-0.26	0.58
		7 Day	-0.39	0.45
	Tgfb1	14 Day	-0.26	0.60
		7 Day	-0.32	0.49
	Tnf	14 Day	-0.41	0.75
		7 Day	0.29	0.69
Degranulation, Compliment	*C3*	14 Day	-0.51	0.83
		7 Day	-0.07	0.27
	Gzmb	14 Day	0.02	1.07
		7 Day	-0.32	0.56
	Prf1	14 Day	-0.29	1.01
		7 Day	-0.17	0.76
Endogenous Control	*18S*	14 Day	0.50	0.34
		7 Day	0.59	0.07
	Actb	14 Day	-0.22	0.13
		7 Day	-0.19	0.08
	Ece1	14 Day	-0.45	0.33
		7 Day	-0.18	0.23
	Edn1	14 Day	-0.49	0.30
		7 Day	-0.33	0.45
	Gapdh	14 Day	-0.33	0.14

		7 Day	-0.42	0.32
	Gusb	14 Day	-0.17	0.33
		7 Day	-0.17	0.27
	Pgk1	14 Day	-0.04	0.19
		7 Day	-0.52	0.26
	Tfrc	14 Day	-0.17	0.11
		7 Day	-0.45	0.10
Enzyme	*Hmox1*	14 Day	-0.38	1.45
		7 Day	-0.48	0.11
	Ptgs2	14 Day	0.04	0.21
		7 Day	-0.36	0.29
Intracellular Signaling	*Agtr2*	14 Day	-0.83	2.74
		7 Day	-1.01	1.67
	Hprt1	14 Day	-0.27	0.15
		7 Day	-0.31	0.09
	Ikbkb	14 Day	-0.30	0.37
		7 Day	-0.03	0.49
	Nfkb1	14 Day	-0.36	0.13
		7 Day	-0.20	0.31
	Nfkb2	14 Day	0.23	0.30
		7 Day	0.20	0.65
	Smad3	14 Day	-0.36	0.31
		7 Day	-0.30	0.21
	Smad7	14 Day	-0.09	0.31
		7 Day	-0.14	0.25
	Socs1	14 Day	0.08	1.00
		7 Day	0.30	0.42
	Socs2	14 Day	-0.22	0.22
		7 Day	-0.37	0.11
	Stat1	14 Day	-0.23	0.82
		7 Day	-0.31	0.47
	Stat3	14 Day	-0.40	0.36
		7 Day	-0.35	0.20
	Stat4	14 Day	-0.39	0.74
		7 Day	0.09	0.51
	Stat6	14 Day	-0.13	0.24
		7 Day	-0.05	0.19
Secreted Factor	*Icos*	14 Day	-0.28	0.91
		7 Day	0.10	0.68
	Nos2	14 Day	0.49	1.00
		7 Day	0.28	2.24
	Vegfa	14 Day	-0.12	0.29
		7 Day	-0.38	0.19
