# Supplementary material for: Environmental Particulate Matter Induces Murine Intestinal Inflammatory Responses and Alters the Gut Microbiome
Source: PLoS One. 2013 Apr 24;8(4):e62220. doi: 10.1371/journal.pone.0062220 (PMC3634745; doi:10.1371/journal.pone.0062220)
Supplement: Table S3 — Effects of 7 and 14 day treatment with PM10 on gene expression in colon in wild-type mice. (PDF) [file pone.0062220.s003.pdf]

Table S3. Effects of 7 and 14 day treatment with PM_{10} on gene expression in colon in wild-type mice.

Class	Gene	Log_2(fold change)		Standard Deviation
Apoptosis	*Bax*	7 Day	-0.33	0.09
		14 Day	-0.01	0.21
	Bcl2	7 Day	-0.06	0.35
		14 Day	-0.13	0.49
	Bcl2l1	7 Day	-0.19	0.41
		14 Day	-0.33	0.25
	Fas	7 Day	-0.13	0.21
		14 Day	-0.12	0.15
	Fasl	7 Day	-0.04	0.37
		14 Day	-0.09	0.40
Cellular Marker	*B2m*	7 Day	-0.28	0.45
		14 Day	-0.54	0.14
	Cd19	7 Day	0.95	1.93
		14 Day	0.57	0.51
	Cd28	7 Day	0.65	0.61
		14 Day	0.18	0.50
	Cd34	7 Day	0.53	0.94
		14 Day	-0.09	0.19
	Cd38	7 Day	-0.77	0.22
		14 Day	-0.01	0.43
	Cd3e	7 Day	-0.68	0.27
		14 Day	-0.24	0.71
	Cd4	7 Day	1.08	0.47
		14 Day	-0.10	0.17
	Cd40	7 Day	0.79	0.91
		14 Day	-0.17	0.50
	Cd40lg	7 Day	1.10	1.52
		14 Day	0.15	1.00
	Cd68	7 Day	0.70	0.75
		14 Day	0.03	0.36
	Cd80	7 Day	1.57	0.36
		14 Day	-0.23	0.20
	Cd86	7 Day	1.04	0.52
		14 Day	-0.04	0.26
	Cd8a	7 Day	-0.59	0.69
		14 Day	0.13	0.51
	Ctla4	7 Day	0.58	0.39
		14 Day	0.01	0.65
	H2-Eb1	7 Day	0.38	0.42
		14 Day	-0.39	0.31
	Il2ra	7 Day	0.61	0.24

		14 Day	0.12	0.31
	Ptprc	7 Day	0.63	0.26
		14 Day	-0.03	0.20
	Ski	7 Day	-0.26	0.26
		7 Day	-0.22	0.40
	Tbx21	14 Day	-1.55	1.20
		7 Day	-0.79	1.25
	Tnfrsf18	14 Day	0.48	0.32
		7 Day	0.01	0.36
Cellular Migration	*Col4a5*	14 Day	0.12	0.43
		7 Day	-0.28	0.38
	Fn1	14 Day	0.85	1.09
		7 Day	0.21	0.31
	Lrp2	14 Day	0.31	0.82
		7 Day	0.57	0.81
	Sele	14 Day	1.22	2.20
		7 Day	0.26	2.26
	Selp	14 Day	0.20	0.98
		7 Day	0.14	0.65
	Vcam1	14 Day	1.14	0.71
		7 Day	-0.14	0.26
Chemokine	*Ccl19*	14 Day	1.01	1.80
		7 Day	-0.59	0.57
	Ccl2	14 Day	0.79	0.86
		7 Day	-0.45	0.45
	Ccl3	14 Day	-0.48	0.77
		7 Day	0.28	0.69
	Ccl5	14 Day	-1.11	0.93
		7 Day	-0.63	0.63
	Ccr2	14 Day	0.19	0.30
		7 Day	0.02	0.32
	Ccr4	14 Day	0.16	0.96
		7 Day	-0.48	0.76
	Ccr7	14 Day	1.80	1.44
		7 Day	-0.21	0.65
	Cxcl10	14 Day	-0.14	0.93
		7 Day	-0.26	0.59
	Cxcl11	14 Day	-2.56	2.44
		7 Day	-0.11	0.33
	Cxcr3	14 Day	-0.64	0.20
		7 Day	-0.15	0.53
Cytokine	*Csf1*	14 Day	0.43	0.58
		7 Day	-0.16	0.50
	Csf2	14 Day	-1.69	0.81
		7 Day	-0.18	0.46
	Ifng	14 Day	0.94	0.87
		7 Day	0.04	0.97

	Il10	14 Day	-1.01	0.15
		7 Day	-0.16	0.55
	Il12a	14 Day	1.38	3.41
		7 Day	2.79	1.71
	Il12b	14 Day	1.01	1.04
		7 Day	-0.39	1.01
	Il13	14 Day	-0.14	3.23
		7 Day	0.16	0.72
	Il15	14 Day	-0.44	0.52
		7 Day	-0.32	0.23
	Il17	14 Day	-1.71	2.86
		7 Day	2.54	1.01
	Il18	14 Day	-0.04	0.45
		7 Day	-0.05	0.13
	Il1a	14 Day	0.37	1.62
		7 Day	-0.62	0.36
	Il1b	14 Day	0.11	0.59
		7 Day	-0.71	0.21
	Il2	14 Day	0.34	0.31
		7 Day	0.78	1.20
	Il4	14 Day	-1.38	2.36
		7 Day	-1.97	2.76
	Il5	14 Day	0.33	0.76
		7 Day	-1.69	1.35
	Il6	14 Day	-0.46	0.35
		7 Day	-0.14	0.31
	Il7	14 Day	0.22	0.26
		7 Day	0.11	0.33
	Tgfb1	14 Day	0.10	0.59
		7 Day	-0.41	0.29
	Tnf	14 Day	-0.61	0.46
		7 Day	-0.14	0.40
Degranulation, Compliment	*C3*	14 Day	0.20	0.27
		7 Day	0.09	0.30
	Gzmb	14 Day	-1.65	1.91
		7 Day	-0.26	0.67
	Prf1	14 Day	0.31	0.73
		7 Day	0.30	0.81
Endogenous Control	*18S*	14 Day	-0.17	0.24
		7 Day	-0.22	0.05
	Actb	14 Day	-0.20	0.29
		7 Day	0.21	0.18
	Ece1	14 Day	-0.71	0.39
		7 Day	0.19	0.35
	Edn1	14 Day	0.17	1.77
		7 Day	0.44	0.64
	Gapdh	14 Day	0.16	0.21

		7 Day	0.02	0.14
	Gusb	14 Day	0.01	0.04
		7 Day	0.20	0.18
	Pgk1	14 Day	0.60	0.46
		7 Day	0.08	0.20
	Tfrc	14 Day	0.66	0.19
		7 Day	0.26	0.22
Enzyme	*Hmox1*	14 Day	-0.64	0.29
		7 Day	-0.09	0.17
	Ptgs2	14 Day	0.47	0.78
		7 Day	0.18	0.32
Intracellular Signaling	*Agtr2*	14 Day	0.91	0.63
		7 Day	-0.81	0.26
	Hprt1	14 Day	-0.09	0.15
		7 Day	-0.10	0.10
	Ikbkb	14 Day	-0.25	0.03
		7 Day	0.14	0.25
	Nfkb1	14 Day	-0.49	0.16
		7 Day	0.09	0.31
	Nfkb2	14 Day	0.03	0.09
		7 Day	-0.19	0.11
	Smad3	14 Day	-0.34	0.22
		7 Day	-0.20	0.35
	Smad7	14 Day	-0.62	0.09
		7 Day	-0.10	0.25
	Socs1	14 Day	-0.63	0.17
		7 Day	0.09	0.47
	Socs2	14 Day	-0.40	0.36
		7 Day	-0.03	0.23
	Stat1	14 Day	-0.44	0.29
		7 Day	-0.09	0.30
	Stat3	14 Day	-0.31	0.30
		7 Day	-0.03	0.37
	Stat4	14 Day	0.67	0.33
		7 Day	0.13	0.13
	Stat6	14 Day	-0.31	0.15
		7 Day	0.03	0.16
Secreted Factor	*Icos*	14 Day	0.89	0.63
		7 Day	0.12	0.43
	Nos2	14 Day	-0.60	0.37
		7 Day	0.44	0.59
	Vegfa	14 Day	0.11	0.30
		7 Day	-0.44	0.28
